# Does war moderate the association between mental wellbeing and its predictors among children? A multi-country cross-sectional study

**DOI:** 10.1186/s12888-025-06795-3

**Published:** 2025-04-29

**Authors:** Shanquan Chen, Sara Rotenberg, Hannah Kuper

**Affiliations:** International Centre for Evidence in Disability, London School of Hygiene & Tropical Medicine, London, WC1E 7HT UK

**Keywords:** War or conflict experience, Mental wellbeing, Children

## Abstract

**Background:**

This study explores how war or conflict influences the established predictors of mental well-being among children, addressing a significant gap in current research.

**Methods:**

Utilizing data from Multiple Indicator Cluster Surveys (MICS6) collected between 2016 and 2021, we examined children aged 5–17 years in four low or middle-low-income countries experiencing war or conflict and compared them to 20 control countries. We employed logistic models to analyze the data, focusing on mental well-being as the outcome. Primary independent variable was exposure to war, with an emphasis on the interaction between this exposure and potential predictors, including age, sex, having a physical disability, enrolled in education, having siblings, living with at least one parent, residence place, and family wealth status.

**Results:**

The analysis revealed significant modifications in the association between factors like age, disability, education, economic status, and place of residence and mental well-being due to war (p values < 0.05). Specifically, the impact of war was more pronounced in older children (OR = 1.48, 95%CI = 1.18–1.85) compared to younger ones. Education was found to mitigate anxiety in conflict-affected areas (OR = 0.75, 95%CI = 0.60–0.95), whereas children with disabilities were more vulnerable to mental health challenges (OR = 2.05, 95%CI = 1.65–2.55) in these settings.

**Limitations:**

The mental well-being measure was based on caregiver reports, which may not fully capture the children’s experiences.

**Conclusion:**

Our findings provide crucial insights into the differential impact of war on children’s mental well-being. They underscore the need for tailored, context-specific mental health interventions for children in conflict-affected areas and encourage further research into the nuanced effects of war on child and adolescent mental health.

**Supplementary Information:**

The online version contains supplementary material available at 10.1186/s12888-025-06795-3.

## Introduction

Children’s mental well-being is influenced by a myriad of factors. Research, predominantly spanning disciplines such as psychology, public health, and sociology, has consistently highlighted the importance of variables such as socioeconomic status, family environment, and educational access [1, 2]. These factors, though crucial, represent a fraction of the broader set of determinants that influence a child’s mental well-being. A context that significantly alters these factors and their impact but remains comparatively under-researched is that of war and conflict [3].

War or conflict situations pose profound challenges to all facets of life, particularly for children, as they drastically change the sociocultural, economic, and environmental. These conditions potentially disrupt and redefine traditional factors impacting mental health, thereby modifying their roles and impacts [4–6]. For example, the protective effect of certain factors, like family environment or socioeconomic status, might weaken or even reverse in the face of war’s hardships [4–6].

Existing literature has provided valuable insights into the mental health outcomes of children in conflict or war settings. Several studies have documented the prevalence of mental health issues among children exposed to war and conflict. For instance, a systematic review by Attanayake et al. (2009) found that the prevalence of post-traumatic stress disorder (PTSD) among children exposed to war ranged from 4.5 to 89.3%, while depression rates ranged from 25 to 25% [7]. Another meta-analysis by Charlson et al. (2019) estimated that in conflict-affected populations, 22.1% of populations had mental disorders, with anxiety and PTSD being the most common [8].

Research has also identified various risk factors for poor mental health outcomes in these settings. Betancourt et al. (2013) found that exposure to violence and loss of caregivers were significant predictors of internalizing problems among war-affected youth in Sierra Leone [9]. Similarly, Panter-Brick et al. (2015) reported that trauma exposure and daily stressors were associated with higher levels of PTSD and depression symptoms among Afghan youth [10].

Moreover, studies have begun to explore protective factors and resilience in children affected by war. Tol et al. (2013) conducted a systematic review of resilience and mental health in children living in areas of armed conflict, finding that factors such as social support, caregiver mental health, and community acceptance were associated with better mental health outcomes [11].

However, despite these valuable contributions, there remains a significant gap in our understanding of how war or conflict interacts with established predictors of children’s mental well-being. Most studies have focused on the direct effects of war exposure on mental health outcomes, but less is known about how war might moderate the association between other factors and mental well-being. This leaves a critical gap in our understanding and capacity to effectively intervene and support the mental health of children living in these extraordinarily challenging contexts.

Building on existing literature and addressing identified research gaps, this study aims to answer three specific questions: (1) How does war or conflict exposure moderate the association between socio-demographic factors and children’s mental well-being? (2) To what extent does it modify the association between family-related factors and mental well-being? (3) How does it alter the association between socio-economic factors and mental well-being? We hypothesize that war or conflict exposure will: H1, amplify the negative association between older age and mental well-being due to increased awareness; H2, strengthen the association between disability status and poor mental well-being due to additional challenges; H3, weaken education’s protective effect on mental well-being due to compromised quality and consistency; H4, enhance the positive association between living with parents and mental well-being due to increased need for stability; and H5, amplify the negative association between lower family wealth status and poor mental well-being due to exacerbated economic hardships. Through these questions and hypotheses, we seek to provide a comprehensive understanding of how war or conflict experiences interact with various aspects of children’s lives to influence their mental well-being.

## Methods

### Data source and participants

We used Multiple Indicator Cluster Surveys (MICS6) data, collected between 2016 and 2021. MICS serves as the predominant dataset employed by United Nations (UN) agencies to evaluate progress towards the accomplishment of Sustainable Development Goals for women and children [12]. Detailed descriptions of MICS, such as sampling methods and quality-control procedures, can be found elsewhere [12, 13].

These analyses were conducted only on samples of children aged 5–17 years. Data from four low or middle-low-income countries (LMICs) (namely the Central African Republic, Democratic Republic of Congo, Nigeria, and the State of Palestine), which were experiencing war or conflict during data collection, were classified as the exposed group. The definition of war or conflict is detailed in the following section. Data from 20 other LMICs (including Bangladesh, Gambia, Georgia, Ghana, Guinea Bissau, Guyana, Honduras, Kiribati, Kyrgyz Republic, Lesotho, Madagascar, Malawi, Nepal, Pakistan, Samoa, Sao Tome and Principe, Togo, Uzbekistan, Vietnam, Zimbabwe) were categorized as control countries.

We excluded the cases with missing values (ranging from 0.01% for having siblings to 17.6% for education) instead of any imputation.

The data are publicly available. The use of secondary de-identified data made this study exempt from institutional review board review. Participants in the original studies gave informed consent and each study was approved by relevant institutional ethics review committees at the country level involved in data collection.

### Outcome and measurement

**Mental well-being** was measured by two questions from the Child Functioning Module (CFM), which was developed by UNICEF and the Washington Group on Disability Statistics and was validated for use in household surveys [14]. Mental well-being was captured by two questions—one on signs of depression (How often does the child seem very sad or depressed? ) and another on anxiety (How often does the child seem very anxious, nervous, or worried? ), with possible responses of “daily,” “weekly,” “monthly,” “a few times a year,” and “never”. Answers with “daily” was defined as being possible depression or anxiety accordingly. For children 5–14, mothers or primary caregivers served as proxy respondents, while children 15–17 answered directly.

### Potential moderator

**War or conflict** in this study was defined based on the Uppsala Conflict Data Program (UCDP) definition. UCDP categorizes armed conflict as “a contested incompatibility that concerns government and/or territory where the use of armed force between two parties, of which at least one is the government of a state, results in at least 25 battle-related deaths in a calendar year“ [15]. The four countries classified as experiencing war or conflict in our study were selected based on three criteria: (1) they were categorized as low or middle-low-income countries (LMICs) according to the World Bank classification during the study period (2016–2021), (2) they experienced active armed conflict, as defined by UCDP, during the data collection period of MICS6 (2016–2021), and (3) they participated in the MICS6 survey, providing necessary data for our analysis.

The selected countries in our study represent diverse conflict situations: the Central African Republic has faced ongoing civil war since 2012 between the Seleka rebel coalition and anti-Balaka militias, resulting in widespread displacement and human rights violations; the Democratic Republic of Congo has experienced prolonged conflict since the 1990s, with armed conflicts persisting particularly in the eastern regions despite the official end of the war in 2003; Nigeria has faced multiple conflicts, including the Boko Haram insurgency since 2009, intercommunal violence, and militancy in the Niger Delta; and the State of Palestine has been involved in one of the world’s longest-running conflicts, characterized by periods of violence, occupation, and political tension. While these countries were all experiencing active conflict during the MICS6 data collection period, we acknowledge that the intensity and nature of conflicts varied. The inclusion of the State of Palestine alongside African countries, despite geographical and historical differences, is justified by our focus on the impact of prolonged conflict on children’s mental well-being across diverse contexts. Our study aims to identify common patterns in how conflict exposure moderates the relationship between various factors and children’s mental well-being, while recognizing the unique aspects of each conflict situation and that war and conflict can manifest differently across these settings.

### Potential predictors

We investigated the following possible commonly cited predictors of mental well-being, extracted from previous literature, including age (years), sex (male vs. female), having a physical disability (yes or no), enrolled in education (yes or no), having siblings (yes or no), living with at least one parents (yes or no), residence place (rural vs. urban), and family wealth status [1, 2]. Family wealth status, a five-category variable, was measured by wealth index (available in the data directly) derived from a principal component analysis based on household assets, characteristics, and infrastructure [16]. Physical disability was measured by questions from CFM, covering domains of vision/seeing, hearing, mobility/walking. Each of these questions had response scales of “no difficulty,” “some difficulty,” “a lot of difficulty,” and “cannot do at all”. Details of the questionnaires can be found elsewhere [13]. Following the definition of UNICEF, children were considered to have a disability if “a lot of difficulty” or “cannot do at all” were reported on at least one of the above domains [14].

### Statistic analysis

To describe the basic characterises, categorical variables were reported as numbers (percentage), and continuous variables were reported as mean (standard deviation, SD).

To investigate if war moderates the association between mental wellbeing and its predictors among children, we fitted data with logistic model. We first fitted full models, with mental well-being (yes or no) as the outcome and war or conflict exposure (yes or no) as the primary independent variable of interest, controlled all potential predictors including age, sex, physical disability, enrolment in education, presence of siblings, living with parents, residence place, and family wealth status, as well as all interaction terms between war or conflict exposure and these potential predictors. These full models were then followed by a step-wise selection procedure based on Akaike Information Criterion (AIC). Survey weighting was used to account for sampling design (including the unequal probability of selection, clustering, and stratification). Odds Ratio (OR) and 95% Confidence Interval (CI) were extracted from the final model.

A sensitivity analysis was conducted by repeating the above analysis with imputation of the missing values instead of omitting them.

Analyses were performed using R (version 4.3.0). Statistical significance was defined as *P* < 0.05. All tests were two-tailed.

## Results

A total of 216,853 children, including 33,090 from countries with war or conflict, were included in the study. Among them, 3.4% showed signs of depression and 5.4% showed signs of anxiety based on responses to a single question for each condition. It’s important to note that these percentages reflect the prevalence of daily experiences of feeling very sad or depressed (for depression) and feeling very anxious, nervous, or worried (for anxiety), as reported by caregivers or the children themselves, rather than clinical diagnoses. A basic description by war or conflict exposure is provided in Table 1.

**Table 1:** Basic description of study sample. Data are shown as mean (standard deviation) or number (%). P values for categorical variables were obtained by t-test and continuous variables by chi-square test

Variables	All	War or conflict exposure (= no)	War or conflict exposure (= yes)	*p* value
Age (years)	10.57 (3.69)	10.58 (3.70)	10.49 (3.65)	< 0.001
Sex (= female)	104,131 (48.0%)	87,989 (47.9%)	16,142 (48.8%)	0.003
Disability (= yes)	18,626 (8.6%)	16,144 (8.8%)	2482 (7.5%)	< 0.001
Enrolled in education (= yes)	194,476 (89.7%)	164,351 (89.4%)	30,125 (91.0%)	< 0.001
Having siblings (= yes)	143,649 (66.2%)	119,146 (64.8%)	24,503 (74.0%)	< 0.001
Wealth status of family				
Lowest	45,753 (21.1%)	39,764 (21.6%)	5989 (18.1%)	< 0.001
2	44,643 (20.6%)	37,730 (20.5%)	6913 (20.9%)	
3	44,973 (20.7%)	37,349 (20.3%)	7624 (23.0%)	
4	42,845 (19.8%)	35,959 (19.6%)	6886 (20.8%)	
Highest	38,639 (17.8%)	32,961 (17.9%)	5678 (17.2%)	
Residence place (= rural)	146,364 (67.5%)	126,608 (68.9%)	19,756 (59.7%)	< 0.001
Living with at last one parents (= yes)	195,487 (90.1%)	166,309 (90.5%)	29,178 (88.2%)	< 0.001
Symptoms of depression (= yes)	7392 (3.4%)	5286 (2.9%)	2106 (6.4%)	< 0.001
Symptoms of anxiety (= yes)	11,621 (5.4%)	8242 (4.5%)	3379 (10.2%)	< 0.001

Among children aged 5–9, there was evidence of significant modification by war or conflict of the association of disability and depression (OR = 1.48, 95%CI = 1.18–1.85), wealth status and depression (ORs < = 0.66, p values < 0.01), and residence place and depression (OR = 1.32, 95%CI = 1.12–1.55); there was also evidence of significant modification by war or conflict of the association of age and anxiety (OR = 1.06, 95%CI = 1.01–1.11), disability and anxiety(OR = 1.62, 95%CI = 1.31-2.00), wealth status and anxiety (ORs < = 0.72, p values < 0.01), and residence place and anxiety (OR = 1.41, 95%CI = 1.22–1.64) (Table 2).

**Table 2:** Moderating role of exposure to war or conflict on the association between mental wellbeing and its predictors among children. Data only show the results of interaction terms. The results are presented as odds ratios (OR) with 95% confidence intervals. The “-” symbol denotes that the corresponding variable was not selected in a step-wise approach, meaning that these factors were not considered relevant in the model based on the data. * *p* < 0.05, ** *p* < 0.01, *** *p* < 0.001

Interaction terms	Children aged 5–9		Adolescent aged 10–17	
	Symptoms of depression (= yes)	Symptoms of anxiety (= yes)	Symptoms of depression (= yes)	Symptoms of anxiety (= yes)
Age (years) x War (= yes)	1.03 (0.98–1.09)	1.06 (1.01–1.11) *	0.98 (0.95–1.01)	0.97 (0.95-1.00)
Sex (= female) x War (= yes)	-	-	-	0.91 (0.81–1.03)
Disability (= yes) x War (= yes)	1.48 (1.18–1.85) ***	1.62 (1.31-2.00) ***	1.13 (0.91–1.41)	1.30 (1.06–1.60) *
Enrolled in education (= yes) x War (= yes)	-	0.84 (0.61–1.17)	0.83 (0.67–1.04)	0.80 (0.66–0.98) *
Having siblings (= yes) x War (= yes)	-	-	0.89 (0.76–1.03)	-
Wealth status of family x War (= yes)				
Lowest	0.57 (0.44–0.74) ***	0.62 (0.48–0.78) ***	0.78 (0.62–0.99) *	0.65 (0.52–0.81) ***
2	0.59 (0.46–0.77) ***	0.59 (0.47–0.75) ***	0.72 (0.57–0.91) **	0.63 (0.51–0.77) ***
3	0.62 (0.48–0.79) ***	0.72 (0.58–0.91) **	0.84 (0.67–1.05)	0.70 (0.57–0.86) ***
4	0.66 (0.51–0.85) **	0.70 (0.56–0.87) **	1.01 (0.81–1.26)	0.82 (0.67–1.01)
Highest	Reference	Reference	Reference	Reference
Residence place (= rural) x War (= yes)	1.32 (1.12–1.55) ***	1.41 (1.22–1.64) ***	1.27 (1.10–1.47) **	1.49 (1.31–1.70) ***
Living with at least one parents (= yes) x War (= yes)	0.87 (0.67–1.13)	0.89 (0.70–1.13)	0.83 (0.68–1.02)	-

Among adolescents aged 10–17, there was evidence of significant modification by war or conflict of the association of wealth status and depression (ORs < = 0.78, p values < 0.05), and residence place and depression (OR = 1.27, 95%CI = 1.10–1.47); there was also evidence of significant modification by war or conflict of the association of disability and anxiety(OR = 1.30, 95%CI = 1.06–1.60), enrolled in education and anxiety (OR = 0.80, 95%CI = 0.66–0.98), wealth status and anxiety (ORs < = 0.70, p values < 0.001), and residence place and anxiety (OR = 1.49, 95%CI = 1.31–1.70) (Table 2).

The interaction terms between sex, having siblings, living with parents, and war, respectively, were not statistically significant (Table 2).

The sensitivity analysis using imputed values instead of omitting them confirmed the consistency of our above results (Sup Table 1).

## Discussion

Here we firstly, to the best of our knowledge, demonstrated that war or conflict experience serves as a moderating variable in the association between age, disability, education, economic status, and place of residence, with mental well-being. Contrarily, this moderating effect does not extend to the associations between gender, the presence of siblings, cohabitation with parents, and mental well-being.

Our analysis revealed that the association between age and anxiety levels in children aged 5–9 was moderated by war or conflict experience. Specifically, we observed a stronger association between older age and anxiety symptoms among children in conflict-affected areas compared to those in non-conflict areas. This finding suggests that older children within this age group may be more likely to report anxiety symptoms in the context of war or conflict, though the cross-sectional nature of our study precludes causal inferences. The exacerbated vulnerability in war or conflict experiences in older children might be attributed to their enhanced cognitive development, enabling a more comprehensive understanding of the situation and its potential dangers [17]. Our study aligns with existing literature, which reinforces the notion that children in conflict zones are at an increased risk of mental health issues [9]. Conversely, no significant interaction was noted in adolescents aged 10–17, echoing findings from a review, which argued that older children might employ more effective coping mechanisms, potentially explaining the observed discrepancy [18].

Our findings also suggested that adolescents enrolled in education within conflict zones had a significantly lower prevalence of symptoms of anxiety than their non-enrolled counterparts. The mitigating effect of education on anxiety might stem from the structure, routine, and support provided by schooling, which could foster resilience in the face of adversities associated with war or conflict [17]. In contrast, this effect was not observed in younger children (aged 5–9), possibly due to their lesser cognitive and emotional maturity to utilize the resources provided within an educational setting in the same way adolescents do. Additionally, the longer exposure to the positive effects of schooling among older children may play a crucial role. Adolescents may have had more time to benefit from the protective factors associated with education, such as social connections, skill development, and a sense of normalcy, which could contribute to their improved ability to manage anxiety in conflict settings. Our findings echo previous works, which underscored the protective role of education for mental health in conflict settings [11, 19]. The absence of a significant interaction in younger children (aged 5–9) highlights the necessity for age-appropriate mental health support and intervention strategies as suggested aforementioned.

Our analysis revealed that both children and adolescents with disabilities demonstrated exacerbated vulnerability to war or conflict experience, and were associated with a higher risk of symptoms indicative of anxiety or depression. These findings keep in line with previous research, which has highlighted the increased vulnerability of children and adolescents in conflict settings to mental health problems [20, 21]. From a practical standpoint, these findings underscore the critical need for focused, inclusive mental health interventions for children and adolescents with disabilities residing in conflict-affected regions. The elevated vulnerability towards war or conflict experiences observed in children and adolescents with disabilities could potentially be attributed to the intersectionality of disability and trauma exposure. The experience of disability may compound the emotional distress caused by war, as individuals with disabilities often face additional societal barriers, stigma, and isolation, which may further exacerbate the psychological impact of conflict [22].

Our study elucidated notable socio-economic and geographical disparities in the prevalence of symptoms of anxiety and depression among children and adolescents residing in conflict zones. We found that less affluent families demonstrated lower prevalence rates, while rural residents exhibited higher rates. The reduced prevalence of anxiety and depression amongst children and adolescents from less affluent families in conflict zones might be attributable to the fact that these groups have developed stronger community bonds and resilience due to their socio-economic conditions, which might buffer against the mental health impacts of war [23]. Nevertheless, the increased prevalence in rural areas may be due to higher exposure to conflict, and fewer resources to cope with the psychological impacts of war [24].

Our findings suggested that the experience of war or conflict did not moderate the associations between gender, the presence of siblings, cohabitation with parents, and mental well-being. This means that irrespective of exposure to conflict, these demographic factors bore a similar association with mental well-being. The consistency of associations between these factors and mental well-being, irrespective of war or conflict experiences, could be due to the universality of these demographic factors as determinants of mental well-being. For instance, gender differences in mental well-being are observed globally, often attributed to biological, social, and cultural factors [25]. Similarly, the presence of siblings and living with parents might influence mental well-being through factors like social support and family dynamics, which are likely to persist regardless of the presence of conflict [26]. Our findings align with global literature emphasizing the importance of these demographic factors for mental well-being [2, 27]. They also echo research in conflict-affected populations indicating the influence of these factors [5, 11, 17].

Our findings have significant implications for policy makers, healthcare providers, and humanitarian organizations working in conflict-affected areas. While several effective interventions already exist, such as school-based programs like Classroom-Based Intervention [28], community-based health services [29], Narrative Exposure Therapy adapted for children (KIDNET) [30], caregiver support programs [31], and art and play therapy [32], our results suggest areas for enhancement. These include developing more inclusive support for children with disabilities, creating age-specific interventions to address the differential impact of war across age groups, strengthening educational programs that show a protective effect for adolescents, extending mental health support to underserved rural areas, and ensuring accessibility across all economic strata. The effectiveness of existing interventions like the IRC’s Families Make the Difference program [31] underscores the potential for positive impact. However, future research should focus on evaluating the long-term effectiveness of these interventions, particularly in addressing the specific vulnerabilities identified in our study, and examining how they can be scaled up and adapted to different cultural contexts within conflict zones.

Our study had important strengths such as the muti-country, large sample and inclusion of a control group. However, the results should be interpreted within the context of several limitations. Firstly, the cross-sectional design precludes examination of changes over time or establishment of causality. Secondly, our binary measure of war or conflict exposure doesn’t account for varying intensity, duration, or types of conflict experiences, nor the time since conflict occurred or whether children are still experiencing war. Thirdly, mental well-being was assessed using only two questions from the Child Functioning Module (CFM), focusing on difficulties managing emotions such as worry, sadness, or anxiety. While validated [14], this simplistic measure doesn’t encompass the full range of possible manifestations of depression or anxiety in young individuals, such as irritability, psychosomatic and sleep disorders, fatigue, and anhedonia. The CFM relies on mothers or primary caregivers as informants rather than directly asking children, which, while partially justified by logistical considerations [14], carries inherent limitations. Parents or caregivers may not be fully aware of the difficulties experienced by the child, particularly regarding “internalising” problems, and there are often disagreements regarding the severity of existing difficulties. This approach may limit comparability and replicability with other studies in the field that use more comprehensive assessment tools, such as the Child PTSD Symptom Scale or the Depression Self-Rating Scale. Our simplified measure, while practical for large-scale surveys, may not capture the full spectrum of mental health issues that children in conflict zones might experience, potentially leading to an underestimation of mental health problems. Nonetheless, the prevalence of daily depression and anxiety signs reported in our sample aligns with global prevalence estimates [14], and the CFM underwent extensive testing to ensure adequate psychometric properties [14]. The brevity of our measure allows for wider implementation across diverse settings, potentially increasing generalizability. Finally, our study did not control for all potential factors, notably omitting maltreatment, bullying, stigma, and parents’ mental health state, despite their documented influence on children’s mental health [33–36], due to the unavailability of this information in the MICS. Further research is needed to address these limitations, particularly through longitudinal studies to establish causal relationships of the dynamic changes we identified for depression/anxiety. Future studies could benefit from incorporating both brief screening tools and more comprehensive assessments to enhance comparability across studies in this field.

In conclusion, our study reveals that war or conflict experiences significantly moderate the associations between several key predictors and children’s mental well-being. Older children (5–9 years) in conflict zones showed increased vulnerability to anxiety, while adolescents enrolled in education demonstrated lower anxiety levels. Both children and adolescents with disabilities exhibited heightened vulnerability to anxiety and depression in conflict settings. Economically, less affluent families in conflict zones showed lower prevalence rates of anxiety and depression symptoms, while rural residents exhibited higher rates. These findings underscore the need for tailored, context-specific interventions, particularly focused on inclusive mental health support for children with disabilities in conflict-affected regions. Educational programs should be maintained and strengthened, especially for adolescents, given their protective effect. Special attention should be directed to rural areas where mental health support may be less accessible, and age-appropriate strategies should be developed to address the differing impacts of conflict across age groups. Our study prompts further investigation into the mechanisms by which war affects children’s and adolescents’ mental well-being so differentially across these factors, calling for longitudinal studies to establish causal relationships and develop targeted interventions. By addressing these critical areas, we can work towards better supporting the mental health and well-being of children and adolescents living in conflict-affected regions.

## Electronic supplementary material

Below is the link to the electronic supplementary material.

Supplementary Material 1

## Data Availability

The data are publicly available (https://mics.unicef.org/surveys). The use of secondary de-identified data made this study exempt from institutional review board review. Participants in the original studies gave informed consent and each study was approved by relevant institutional ethics review committees at the country level involved in data collection.
