# Supplementary material for: Does war moderate the association between mental wellbeing and its predictors among children? A multi-country cross-sectional study
Source: BMC Psychiatry. 2025 Apr 29;25:436. doi: 10.1186/s12888-025-06795-3 (PMC12042364; doi:10.1186/s12888-025-06795-3)
Supplement: Supplementary file 1 — Supplementary Material 1 [file 12888_2025_6795_MOESM1_ESM.docx]

**Sup Table 1. Moderating role of exposure to war or conflict on the association between mental wellbeing and its predictors among children, imputing on missing values.** Data only show the results of interaction terms. The results are presented as odds ratios (OR) with 95% confidence intervals. The "-" symbol denotes that the corresponding variable was not selected in a step-wise approach, meaning that these factors were not considered relevant in the model based on the data. * p < 0.05, ** p < 0.01, *** p < 0.001

| **Interaction terms** | **Children aged 5-9** | | **Adolescent aged 10-17** | |
| --- | --- | --- | --- | --- |
|  | **Symptoms of depression (=yes)** | **Symptoms of anxiety (=yes)** | **Symptoms of depression (=yes)** | **Symptoms of anxiety (=yes)** |
| **Age (years)** x **War (=yes)** | 1.01 (0.98-1.05) | **1.05 (1.00-1.10) *** | 0.99 (0.96-1.03) | 0.99 (0.97-1.01) |
| **Sex (=female)** x **War (=yes)** | - | - | - | 0.97 (0.78-1.21) |
| **Disability (=yes)** x **War (=yes)** | **1.56 (1.42-1.72) ***** | **1.79 (1.63-1.96) ***** | 1.37 (0.93-2.03) | **1.56 (1.42-1.72) ***** |
| **Enrolled in education (= yes)** x **War (=yes)** | - | 0.86 (0.54-1.37) | 0.82 (0.57-1.19) | **0.61 (0.40-0.92) *** |
| **Having siblings (= yes)** x **War (=yes)** | - | - | 0.71 (0.42-1.22) | - |
| **Wealth status of family** x **War (=yes)** |  |  |  |  |
| Lowest | **0.52 (0.4-0.67) ***** | **0.58 (0.46-0.74) ***** | **0.84 (0.67-1.07) *** | **0.62 (0.5-0.78) ***** |
| 2 | **0.65 (0.5-0.84) ***** | **0.53 (0.42-0.67) ***** | **0.66 (0.53-0.84) **** | **0.67 (0.54-0.82) ***** |
| 3 | **0.61 (0.47-0.78) ***** | **0.65 (0.52-0.82) **** | 0.89 (0.71-1.12) | **0.65 (0.53-0.79) ***** |
| 4 | **0.65 (0.5-0.84) **** | **0.69 (0.55-0.86) **** | 1.07 (0.86-1.34) | 0.89 (0.72-1.09) |
| Highest | Reference | Reference | Reference | Reference |
| **Residence place (=rural)** x **War (=yes)** | **1.29 (1.1-1.52) ***** | **1.5 (1.29-1.74) ***** | **1.38 (1.19-1.59) **** | **1.52 (1.33-1.73) ***** |
| **Living with at least one parents (= yes)** x **War (=yes)** | 0.96 (0.74-1.25) | 0.98 (0.77-1.25) | 0.9 (0.73-1.1) | - |
